# Occult Hepatitis B Virus Infection in Chacma Baboons, South Africa

**DOI:** 10.3201/eid1904.121107

**Published:** 2013-04

**Authors:** Caroline Dickens, Michael C. Kew, Robert H. Purcell, Anna Kramvis

**Affiliations:** University of the Witwatersrand, Johannesburg, South Africa (C. Dickens, M.C. Kew, A. Kramvis);; Groote Schuur Hospital, Cape Town, South Africa (M.C. Kew);; National Institutes of Health, Bethesda, Maryland, USA (R.H. Purcell)

**Keywords:** hepatitis B virus, viruses, nonhuman primate, genotypes, transmission, silent infection, occult infection, Chacma baboon, Papio ursinus orientalis, zoonoses, South Africa

## Abstract

During previous studies of susceptibility to hepatitis B virus (HBV) infection, HBV DNA was detected in 2/6 wild-caught baboons. In the present study, HBV DNA was amplified from 15/69 wild-caught baboons. All animals were negative for HBV surface antigen and antibody against HBV core antigen. Liver tissue from 1 baboon was immunohistochemically negative for HBV surface antigen but positive for HBV core antigen. The complete HBV genome of an isolate from this liver clustered with subgenotype A2. Reverse transcription PCR of liver RNA amplified virus precore and surface protein genes, indicating replication of virus in baboon liver tissue. Four experimentally naive baboons were injected with serum from HBV DNA–positive baboons. These 4 baboons showed transient seroconversion, and HBV DNA was amplified from serum at various times after infection. The presence of HBV DNA at relatively low levels and in the absence of serologic markers in the baboon, a nonhuman primate, indicates an occult infection.

Hepatitis B virus (HBV) is a 3.2-kb partially double-stranded virus belonging to the family *Hepadnaviridae*. The outcome of infection with this virus is determined mainly by the immune response of the host and can be acute, chronic, or occult. HBV is divided into 9 genotypes (A–I); an additional genotype, J, has also been proposed (*1*–*3*). Several genotypes are further divided into subgenotypes. In sub-Saharan Africa, subgenotypes A1 and D3 and genotype E circulate (*4*).

Hepadnaviruses can infect avian and mammalian hosts but have a limited host range, infecting only their natural hosts and a few closely related species. Naturally occurring infections have been found in several Old and New World nonhuman primates, such as chimpanzees (*5*), gorillas (*6*), gibbons (*7*), orangutans (*8*) and woolly monkeys (*9*). HBV, whose natural host is humans, also infects chimpanzees (*10*); Barbary macaques (*11*); and tree shrews (*12*), in addition to humans.

Baboons (*Papio* species) have been proposed as a possible animal model of HBV infection. Phylogenetically, baboons are close to humans, showing ≈96% homology at the DNA level, and they have an immune system similar to that of humans (*13*). Early studies involving injection of baboons with HBV-positive serum failed to detect any clinical or biochemical signs of infection in these primates, and initial serologic surveys failed to detect HBV surface antigen (HBsAg) in serum, leading to the conclusion that baboons were not susceptible to HBV infection (*14*). This supposed lack of susceptibility of baboons to infection with HBV, and the fact that unlike chimpanzees, baboons are not an endangered species, intimated that baboons were good candidates for sources of liver for xenotransplants. The use of xenotransplants from pigs and nonhuman primates to humans was considered to overcome the donor shortage and to bridge patients with terminal hepatic failure until a human donor organ became available (*15*).

To confirm that baboons were not susceptible to HBV infection, Kedda et al. injected 6 wild-caught Chacma baboons (*Papio ursinus orientalis*) with pooled HBV-positive serum and analyzed the baboons for 52 weeks by using sensitive molecular techniques to detect evidence of transmission (*16*). HBV DNA was detected by nested PCR in serum and liver of 4 of the baboons <52 weeks after injection. Liver function and histologic results were within reference ranges, and HBsAg was not detected in serum (*16*). However, during that study, HBV DNA was detected by using nested PCR in serum of 2 of the 6 baboons at baseline, before injection with HBV. The presence of HBV DNA was confirmed by retesting samples in independent laboratories. This finding raised the possibility that baboons were naturally infected with a hepadnavirus. The aims of the present study were to determine the prevalence of HBV in wild baboons, molecularly characterize the virus isolated from these baboons, determine whether the virus replicates in the baboon liver, and demonstrate viral transmission to experimentally naive baboons.

## Methods

Serum samples obtained from 49 adult and 20 juvenile Chacma baboons caught in the Western Cape, Eastern Cape, and Limpopo Provinces of South Africa were stored at −70°C. Liver tissue (fresh frozen and formalin-fixed) was obtained from 1 of the adult wild-caught baboons (hereinafter referred to as baboon 9732), which was euthanized for medical and ethical reasons. Permission for this study was obtained from the Animal Ethics Committee of the University of the Witwatersrand. All procedures were approved by this Committee. Baboons were provided care according to the guidelines of the South African Medical Research Council.

### Serologic and Immunohistochemical Analysis

Baboon serum samples were tested for HBsAg and for antibodies against HBV core antigen (anti-HBc) by using commercially available assays (Abbott Laboratories, Abbott Park, IL, USA). Alanine and aspartate aminotransferase levels were measured by using a 747 Automatic Analyzer (Hitachi, Tokyo, Japan).

Formalin-fixed liver tissue from baboon 9732 was used for histologic and immunohistologic preparations. Liver tissue was embedded in paraffin and sectioned. The sections were stained with hematoxylin and eosin for histologic examination or with immunoperoxidase and polyclonal antibodies to HBsAg and HBV core antigen (HBcAg) to detect HBsAg and HBcAg in hepatocytes.

### Southern Hybridization Slot Blot

Baboon serum samples were blotted onto a nylon membrane by using a slot-blot manifold according to the protocol described by Zaaiger et al., which has a detection limit of 2.5 × 10^7^ HBV genomes/mL (*17*). HBV DNA was detected by Southern blot hybridization with a ^32^P-labeled HBV DNA probe (HBV DNA in pBV325 vector, *adr* subtype).

### DNA Extraction and Amplification and Phylogenetic Analysis

DNA was extracted from 200 μL of baboon serum by using the QIAamp DNA Blood Mini Kit (QIAGEN, Hilden, Germany) according to the manufacturer’s instructions. DNA was also extracted from baboon liver tissue by using a phenol-chloroform extraction method (*18*). Extracted DNA was quantified by using spectrophotometric analysis with a NanoDrop ND-1000 Spectrophotometer (NanoDrop Technologies Inc., Wilmington, DE, USA).

Subgenomic nested PCR amplifications of the precore/core (nt 1732–2045 and nt 1765–1968), core (nt 1687–2498 and nt 2267–2436), polymerase (nt 2540–2896 and nt 2566–2858), and surface (nt 255–759 and nt 459–710) regions were used to confirm the presence of HBV DNA in the serum extracts by using 40 cycles (*16*). The sensitivity of these amplifications is 40–400 HBV genomes/mL (*19*).

**Figure 1:**
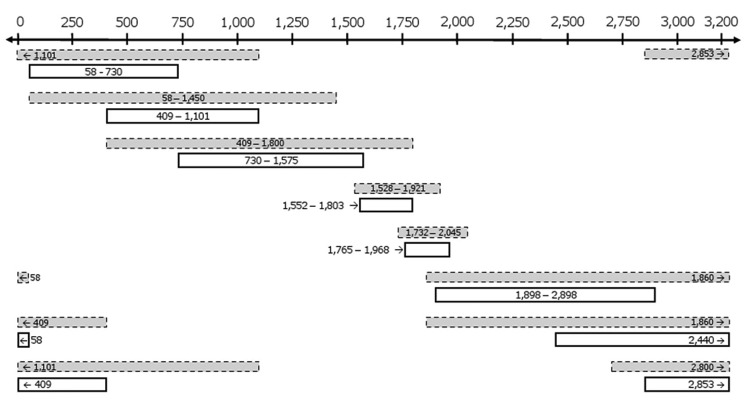
Amplification of the hepatitis B virus HBV genome by using overlapping subgenomic fragments. Shown are 8 overlapping subgenomic fragments amplified by nested PCR, These fragments were used to generate the complete HBV sequence isolated from liver tissue of Chacma baboon 9732, South Africa. Dashed gray boxes indicate first-round PCRs, and white boxes indicate second-round PCRs. Values along the 2-headed arrow at the top are in basepairs from the *Eco*RI site of the circular genome of HBV. Small arrows within boxes indicate the direction of amplification.

The complete viral genome was amplified by using 8 overlapping subgenomic fragments (Figure 1). Thermocycling conditions and sequences for primers 58, 409, 730, 1101, 1450, 1860, 2440, and 2853 were obtained from Hu et al. (*20*). Additional primers used were 1575 (5′-CCGGCAGATGAGAAGGCACAGACGG-3′), 1528 (5′-ACCTCTCTTTACGCGGTCTC-3′), 1552 (5′-TCTGTGCCTTCTCATCTGCC-3′), 1800 (5′-AGACCAATTTATGCCTACAGCCTCCTA-3′), 1803 (5′-CGCAGACCAATTTATGCCTAC–3′), 1898 (5′-GGCATGGACATTGACCCGTA-3′), 1921 (5′-TTTATACGGGTCAATGTC-3′), 2800 (5′-CAGGTAGCGCCTCATTTTGTGGGTCACCATATTCT-3′), and 2898 (5′-GAGGATTGGGAACAGAAAGATT-3′). All nucleotide numbering refers to the position from the *Eco*RI site as position 1.

Amplicons were sequenced directly by using the BigDye Terminator version 3.1 Cycle Sequencing Ready Reaction Kit (Applied Biosystems., Foster City, CA, USA) and sequenced on an ABI3130*xl* Genetic Analyzer with 16 capillaries (Applied Biosystems) and the same primers that were used for amplification. The sequence has been deposited in GenBank under accession no. JX507080. The HBV genomic sequence obtained from the baboon was compared with corresponding sequences of HBV from GenBank as described (*4*).

### RNA Extraction, Reverse Transcription, and Amplification

RNA was extracted from varying amounts of baboon liver tissue by using the guanidinium-acid-phenol method (*21*) and digested with RNase-free DNase I (Fermentas, Waltham, MA, USA) to remove any contaminating DNA. The RNA concentration was determined by spectrophotometry with the NanoDrop ND-1000 Spectrophotometer. cDNA was generated by using SuperScript III reverse transcriptase (Invitrogen, Carlsbad, CA, USA) and oligo(dT)_18_ primers (Invitrogen) in accordance with the manufacturer’s instructions. Non–reverse transcribed negative controls were prepared in an identical manner except that diethylopyrocarbonate–treated water (Invitrogen) was added to each reaction instead of reverse transcriptase. The success of the reverse transcription reaction was confirmed by PCR amplification of a portion of the glyceraldehyde-3-phosphate dehydrogenase gene (*22*). Subgenomic nested PCR amplifications of the precore/core (nt 1732–2045 and nt 1765–1968) and surface (nt 255–759 and nt 459–710) regions were also performed.

### Detection of Covalently Closed Circular DNA

DNA was extracted from baboon liver tissue by using the QIAamp DNA Mini Kit (QIAGEN). DNA extracts were treated with Plasmid-Safe ATP-Dependent DNase (Epicenter Biotechnologies, Madison, WI, USA) to selectively hydrolyze linear double-stranded chromosomal DNA while leaving HBV covalently closed circular DNA (cccDNA) intact. The cccDNA was detected by real-time PCR (*23*) with the Power SYBR Green PCR Master Mix (Applied Biosystems).

### Transmission of HBV to Experimentally Naive Baboons

Transmission of HBV to experimentally naive baboons was performed at the National Institutes of Health (Bethesda, MD, USA). Animals were housed and maintained at Bioqual, Inc. (Rockville, MD, USA). Housing and care of animals complied with all relevant guidelines and requirements, and the animals were housed in facilities that are fully accredited by the Association for Assessment and Accreditation of Laboratory Animal Care International. All protocols were reviewed and approved by the Institutional Animal Care and Use Committees of the National Institute of Allergy and Infectious Diseases of the National Institutes of Health and Bioqual, Inc. Experimentally naïve, domestically raised baboons were obtained from a domestic breeder (Mannheimer Foundation, Homestead, FL, USA). Before inclusion of animals in the study, serum was free of all markers of HBV replication when tested serologically and by nested PCR as described above.

Four experimentally naive baboons were each inoculated with 500 μL of serum obtained from HBV DNA–positive wild-caught baboons from South Africa. Each baboon was injected with serum from a single wild-caught baboon. After injection, serum was obtained from each of the 4 newly injected baboons at weekly intervals. Serum was used to measure levels of alanine aminotransferase, isocitrate dehydrogenase, γ-glutamyltranspeptidase, HBsAg, HBV e antigen, antibodies against HBV e antigen, antibodies against HBsAg, and anti-HBc.

DNA extracted from serum samples was used for nested PCR amplification of a region of the surface gene (nt 459–710). Twenty-five weeks after injection, the study was terminated and the baboons were euthanized. At necropsy, liver tissue and serum was obtained from each baboon for further testing to confirm that virus extracted from experimentally naive baboons was the same as that found in the original baboons.

## Results

### Prevalence of HBV in Wild-caught Baboons in South Africa

The prevalence of HBV in the baboon (*P. ursinus orientalis*) population in South Africa was determined by extracting DNA from serum of 69 wild-caught baboons and amplifying 4 regions of the viral genome (the precore/core, core, polymerase, and surface regions) by nested PCR. HBV DNA could not be amplified with a single-round PCR, indicating that HBV DNA was present at low levels in baboon serum.

Using the criterion of >3 of the 4 regions being PCR positive, we found that 11 (22.4%) of 49 adult and 4 (20.0%) of 20 juvenile wild-caught baboons were positive for HBV. The overall prevalence of hepadnaviral DNA in baboons was 21.7% (15/69). Furthermore, the presence and specificity of the HBV DNA was confirmed when detected directly in the serum of 5 of the 69 baboons by using Southern blot analysis. Only 5 of the 15 PCR-positive serum samples were positive by Southern hybridization because of the relatively lower sensitivity of this method.

### Serologic, Liver Function, and Immunohistologic Tests

**Figure 2:**
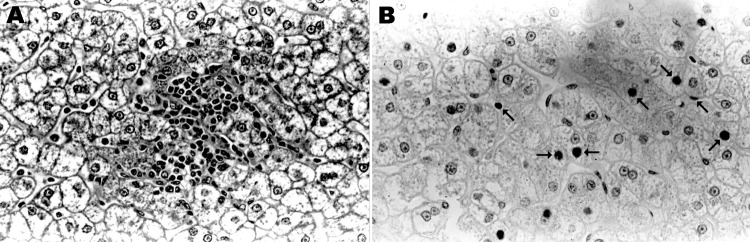
Liver tissue from Chacma baboon 9732, South Africa, showing lobular hepatitis. Liver tissue was obtained at necropsy, fixed in formalin, embedded in paraffin, and sectioned. A) Hematoxylin and eosin staining, showing a focus of mild lobular hepatitis but no evidence of interface hepatitis or bridging necrosis. Portal tracts are normal. B) Immunoperoxidase staining with polyclonal antibody against hepatitis B core antigen. Core antigen was detected in the occasional hepatocyte nucleus. Arrows indicate selected positive nuclei. Original magnifications ×400.

Serum samples from 4 of the 15 HBV DNA–positive baboons, for which additional serum was available, were tested and found to be negative for HBsAg and anti-HBc. Alanine and aspartate aminotransferase levels were within reference ranges, and after high-speed centrifugation and treatment with antibody against HBsAg, no viral particles were observed in the serum by electron microscopy. Histologic examination of liver tissue from 1 of these baboons (9732) showed mild focal lobular hepatitis but no evidence of interface hepatitis, bridging necrosis, dysplasia of hepatocytes, cirrhosis, or hepatocellular carcinoma (Figure 2, panel A). Immunohistochemical staining of liver tissue showed HBcAg in nuclei of some hepatocytes with a patchy distribution (Figure 2, panel B). HBsAg and 42-nm enveloped (Dane) particles were not detected in the cytoplasm.

### Amplification, Sequencing, and Phylogenetic Analysis of HBV Genome from Baboon Liver

The low viral loads indicated that the complete HBV genome could only be amplified subgenomically and because of the small volumes of serum available, liver tissue obtained from baboon 9732 was used to further characterize HBV in baboons. The complete HBV genome was amplified by nested PCR of 8 overlapping subgenomic fragments (Figure 1).

**Figure 3:**
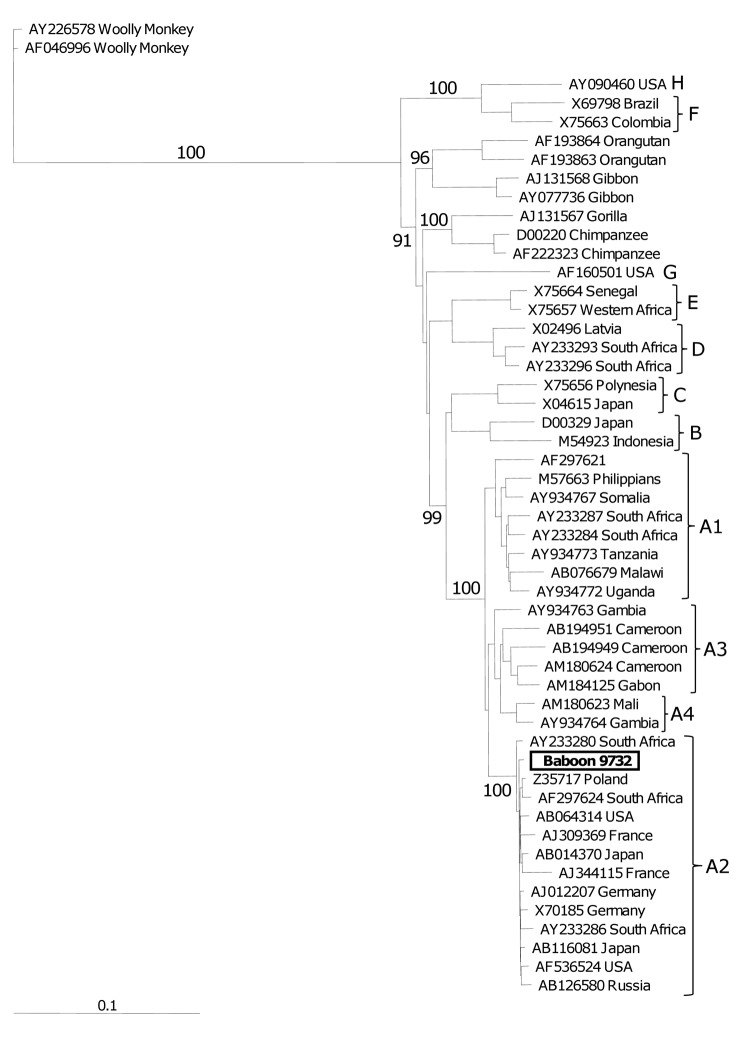
Dendogram of the complete hepatitis B virus (HBV) genome isolated from Chacma baboon 9732, South Africa. Samples representative of all 8 HBV genotypes and primate hepadnaviruses are included. Samples are numbered according to their GenBank accession numbers, followed by their country of origin. The sample from baboon 9732 (**boldface**) clusters strongly with the subgenotype A2 isolates (bootstrap value 100). Letters along the right indicate genotypes. Values along branches are bootstrap values. Scale bar indicates nucleotide substitutions per site.

Phylogenetic analyses of the complete genome showed that HBV from the baboon was closely related to HBV subgenotype A2 (Figure 3). This finding was confirmed by comparison of mean ± SD nucleotide divergence calculations compared to subgenotype A2 (1.00 ± 0.55) and to subgenotype A1 (4.52 ± 0.42). Similar results were obtained for each of the 4 open reading frames. The baboon HBV had mutations in the basic core promoter and precore regions not found in subgenotype A2. These mutations included the G1809T/C1812T double mutation in the Kozak sequence preceding the precore protein start codon and G1888A in the precore region. G1809T/C1812T mutations are characteristic of subgenotype A1, and G1888A is unique to subgenotype A1 (*4*,*24*). Translation of the 4 open reading frames showed them to be well conserved relative to the consensus sequence of subgenotype A2 with the following exceptions: T380C resulting in an rtV84A in conserved region A of the polymerase and a C76R in HBsAg; A2019G resulting in an E40G in the core protein; and C1470T resulting in a P33G in the X protein and T1765C resulting in a P145S in the X protein.

### Expression of HBV RNA in Baboon Liver Tissue

**Figure 4:**
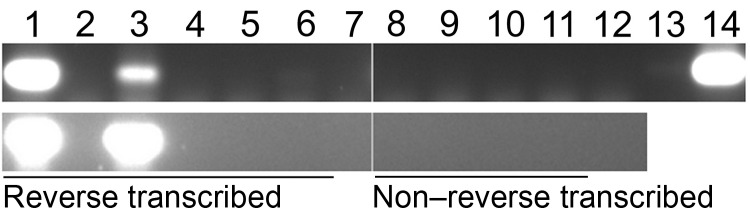
Nested PCR amplification of a subgenomic region of cDNA for baboon hepatitis B virus, South Africa. Reverse transcribed, DNase I–treated cDNA products were amplified by PCR, and amplicons were resolved by electrophoresis on a 1% agarose gel containing ethidium bromide. Non–reverse transcribed samples (in which diethyl pyrocarbonate [DEPC]–treated water was added instead of enzyme during reverse transcription) were included as negative controls. Top panel: Nested PCR 1: 255F–759R; PCR 2: 459F–710R. Bottom panel, 550-bp region of glyceraldehyde-3-phosphate dehydrogenase gene amplified to assess quality of mRNA. Lanes 1 and 8, 100 mg of baboon liver tissue used for RNA extraction; lanes 2 and 9, RNA extraction negative control; lanes 3 and 10, 200 mg of baboon liver tissue used for RNA extraction; lanes 4 and 11, RNA extraction negative control; lane 5, DNase I treatment negative control in which DEPC-treated water was added instead of RNA; lane 6, reverse transcription negative control in which DEPC-treated water was added instead of cDNA; lanes 7 and 12, double-round nested PCR negative control containing best-quality water instead of cDNA; lane 13, single-round PCR negative control containing best-quality water instead of cDNA; lane 14, PCR-positive control containing DNA extracted from liver tissue of baboon 9732 as template.

RNA extracted from liver tissue of baboon 9732 was reverse transcribed and amplified in precore/core (nt 1765–1968) and surface (nt 459–710) open reading frames (Figure 4). Sequences of these amplicons were identical to sequences of the DNA isolated from liver of baboon 9732.

### Identification of cccDNA in Baboon Liver Tissue

cccDNA was detected in liver tissue of baboon 9732 by using real-time PCR. DNA from HBV DNA–positive tumorous and nontumorous human liver and plasmid DNA containing a greater than full-length HBV genome were used as positive controls, and DNA from rat liver tissue was used as a negative control. HBV cccDNA–positive controls had melting temperatures of 82.7°C–84.0°C, and negative controls had melting temperatures <80.0°C. Samples from the baboon liver tissue showed similar melting temperatures as positive controls (82.7°C–83.1°C) indicating that HBV cccDNA was present.

### Transmission of HBV from Wild-caught Baboons to Experimentally Naive Baboons

**Figure 5:**
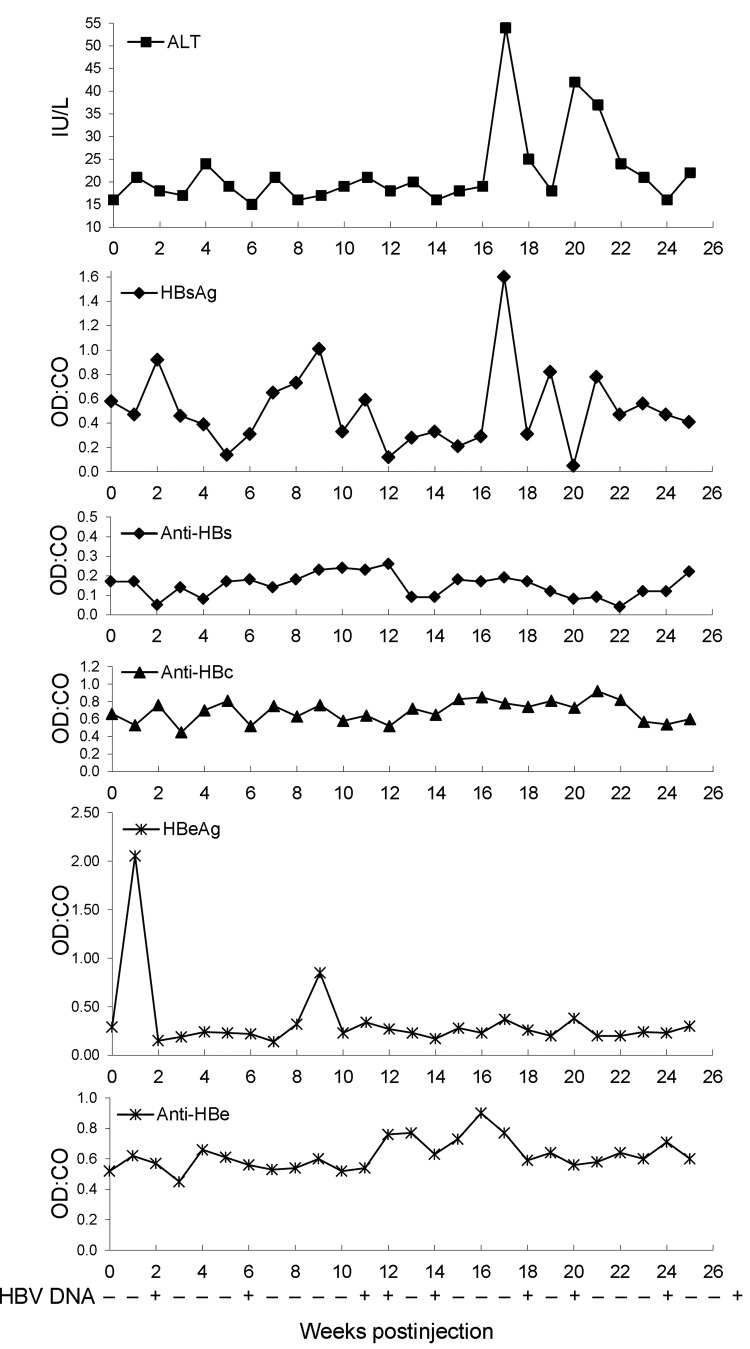
Levels of alanine aminotransferase (ALT) and hepatitis B virus (HBV) serologic markers and detection of HBV DNA in baboon 2. Serum obtained from baboon 2 (which was injected with serum from baboon 9732), and ALT and HBV serologic marker levels were measured at weekly intervals after injection. Serum for week 0 levels was obtained just before injection. OD:CO indicates optical density:cutoff value ratios. An OD:CO >1 indicates a positive result. HBV DNA was detected by nested PCR amplification (255–761 in the first round and 459–710 in the second round) of a 252-bp region of the virus surface gene by using DNA extracted from serum obtained from the infected baboon at weekly intervals. HBsAg, HBV surface antigen; Anti-HBs, antibody against HBV surface antigen; Anti-HBc, antibody against HBV core antigen; HBeAg, HBV e antigen; Anti-HBe, antibody against HBV e antigen. + indicates that the sample amplified successfully at this time point, and – indicates no amplification of virus DNA. The week 26 time point indicates the result of amplification by using DNA extracted from serum at necropsy, and the final time point indicates successful amplification of the virus product from DNA extracted from liver tissue obtained from baboon 2 at necropsy.

Each of 4 experimentally naive, domestically raised baboons was injected with serum from 1 of 4 wild-caught HBV DNA–positive baboons. Serum samples from the experimentally naive baboons were HBV DNA negative before injection, and baseline levels of aspartate aminotransferase, alanine aminotransferase, and IgG were also determined. The baboons were transiently positive for several of these markers and were intermittently positive for HBV DNA in serum. Results for a representative sample (baboon 2) infected with serum from baboon 9732 are shown in Figure 5. DNA extracted from liver tissue obtained at necropsy from baboon 2 was used to amplify and sequence a portion of the surface gene (nt 409–1101). The sequence of virus extracted from liver tissue of baboon 2 six months after injection was identical to the HBV sequence of this region found in original baboon 9732.

## Discussion

Detection of HBV DNA in serum samples of 2 Chacma baboons before injection with human HBV (*16*) suggested that baboons might be chronically infected with HBV. Our objective was to determine the prevalence of HBV in wild-caught Chacma baboons and to characterize the virus isolated from these animals. The overall prevalence (21.7%) of HBV in the baboons is similar to the HBV prevalence in other nonhuman primates in areas to which HBV is highly endemic, including sub-Saharan Africa (*25*).

The baboons were serologically negative, and the 1 baboon examined histologically had mild focal lobular hepatitis, and HBcAg was detected in liver tissue by immunohistochemical staining. Detection of low levels of HBV DNA in baboon liver tissue and in serum in the absence of HBsAg and at relatively low levels of HBV DNA classifies this infection as occult (*26*). Lack of any serologic markers of HBV infection further distinguishes this infection as a seronegative occult HBV infection (*26*). This finding is analogous to a secondary occult infection in the woodchuck (*27*). Furthermore, HBV DNA was detected in juvenile and adult baboons, suggesting lifelong persistence of the virus, which was successfully transmitted to experimentally naive baboons. Detection of HBV DNA alone does not necessarily correspond to an HBV infection (*28*). Thus, detection of cccDNA and viral RNA in liver tissue of baboon 9732 showed that HBV was replicating. The low levels of viral nucleic acids detected in baboon liver are a further characteristic of occult infections (*27*). This study demonstrates a naturally occurring occult HBV infection in a nonhuman primate.

Phylogenetic analysis of the baboon HBV genome showed that it belonged to genotype A, clustering with subgenotype A2. This finding is an unexpected result because subgenotype A1 predominates in South Africa (*24*). However, in the basal core promoter/precore region, the baboon HBV had distinct characteristics of subgenotype A1. Four additional mutations in the polymerase, surface, X, and core regions of the baboon HBV strain differentiated the baboon isolate from most subgenotype A2 isolates. These mutations are not known to cause any major functional or conformational changes.

Paradoxical identification of subgenotype A2 in the baboon when subgenotype A1 predominates in Africa might indicate that subgenotype A2 is an older strain that previously circulated in Africa, which has been replaced by other strains, including subgenotype A1 and genotypes D and E (*29*). An analogous trend might have occurred in the Mediterranean region where genotype D now predominates over genotype A (*30*). Similarly, a change in the prevalent HBV genotype in central and western Africa has been postulated to have occurred over the past 200 years, and genotype E, originally restricted to the west coast of Africa, now spreads over a large crescent stretching from The Gambia, through Nigeria and the Democratic Republic of the Congo into Namibia and Mozambique (*31*). There is a paucity of sequencing data for subgenotype A2 from Africa. Only 4 complete subgenotype A2 genomes from South Africa have been deposited in GenBank. Shorter subgenomic sequences of subgenotype A2 from South Africa (*32*), Tunisia (*33*), and Kenya (*34*) have also been deposited. More extensive molecular epidemiologic studies in Africa might uncover a higher circulation of subgenotype A2 in more remote regions.

Another explanation for the paradoxical finding of subgenotype A2 in the baboon could be that in Africa, as shown in India, subgenotype A2 isolates are confined to peripheral blood leukocytes (PBLs). In India, subgenotype A1 and genotype D circulate, but actively replicating HBV subgenotype A2 isolates have been detected in PBLs (*35*). These subgenotype A2 isolates were confined to PBLs and were not detected in serum. Differential immune pressures are believed to compartmentalize HBV to different parts of the body in which different strains evolve independently (*35*). Further study will be needed to determine whether subgenotype A2 is compartmentalized to PBLs in Africa.

In sub-Saharan Africa, most human HBV carriers are negative for HBV e antigen, and in these persons, spread of HBV is mainly horizontal (*36*). In baboons, their natural habits indicate that horizontal baboon-to-baboon transmission of HBV is highly probable. Baboons are social animals, and bonds are strengthened by daily grooming with several related and unrelated partners, including offspring (*37*). Young baboons, like their human counterparts, spend much of the day playing together. The games can become quite physical, often leading to mock fights. Physical fights among male and female adult baboons are rare. Alpha males tend to be aggressive, and displays of dominance and chases occur daily.

The well-documented interactions between humans and baboons make cross-species transmission of this virus extremely plausible (*37*). Baboons are the most widely distributed nonhuman primates in Africa and are found in virtually all parts of sub-Saharan Africa. They often appear in ancient Egyptian mythology and art, depicted as captives brought from southern Africa or as pets on leashes (*37*). In southern Africa, baboons have been kept as pets and trained to work as oxcart drivers, railway laborers, and as goat herders on farms (*37*). However, more common are reports of conflict between humans and baboons. In the rural areas, baboons raid orchards, destroy irrigation pipes, and kill sheep and goats. Because baboons can become aggressive when challenged, they are often killed by farmers for being pests. Slaughter of baboons for bush meat could be another source of exposure to the virus (*38*).

From the results of the present study, it is impossible to determine whether baboons were infected with HBV by humans, as has been hypothesized (*39*), or whether humans were infected by baboons. However, as noted by Michael Lai, a virus expert, “When we expose ourselves to exotic animals, there is always a risk of being exposed to something unknown…When we perturb the existing peace between human beings and nature, we are opening a Pandora’s box, which may contain surprises” (*40*).
